# Prognostic role of immune environment in luminal B early breast cancer

**DOI:** 10.1002/cam4.5642

**Published:** 2023-02-07

**Authors:** Arlene Chan, Jespal Gill, HuiJun Chih, Delia J. Nelson

**Affiliations:** ^1^ Curtin Medical School Curtin University Perth WA Australia; ^2^ Breast Cancer Research Centre‐WA, Hollywood Private Hospital Nedlands WA Australia; ^3^ Western Diagnostics Pathology Perth WA Australia; ^4^ Curtin School of Population Health Perth WA Australia; ^5^ CHIRI Biosciences Curtin University Perth WA Australia

**Keywords:** anti‐cancer immunity, checkpoint molecules, luminal B breast cancer, predictive, prognostic

## Abstract

The importance of the immune microenvironment in triple negative and HER2‐amplified breast cancer (BC) is well‐established; less is known about the immune environment in luminal breast cancers. We aimed to assess for the impact of immune biomarkers on BC outcome in a group of luminal B patients with archived tissue and annotated clinical information. Patients with early breast cancer (EBC) treated in a single institution over a 14‐year period, with prospectively collected data were included. Luminal B EBC patients were identified and defined into three cohorts: A: grade 2 or 3, ER & PR positive, HER2‐negative; B: Any grade, ER positive, PR and HER2‐negative (Ki67 ≥ 14% in cohorts A & B); and C: Any grade, ER or PR positive, HER2‐positive. Within each cohort, patients with a relapsed BC event (R) were compared on a 1:1 basis with a control patient (C) who remained disease‐free, balanced for key characteristics in an effort to balance the contribution of each clinical group to outcome. Archival breast, involved and uninvolved axillary nodes were assessed by immunohistochemistry for biomarkers identifying effector and suppressor immune cells, and compared between R and C. In total, 120 patients were included (80, 22, and 18 patients in cohorts A, B, and C, respectively). R were 1.5 years older (*p* = 0.016), with all other characteristics being balanced. Overall, there were no statistically significant differences in immune biomarkers in breast or nodal tissue of R and C. However, there was a trend toward higher levels of TILs in breast tumors of C, while GAL‐9 was consistently expressed on lymphocytes and tumor cells in all breast and nodes of C and was absent from all tissues of R. These trends in checkpoint molecule expression deserve further research.

## Graphical abstract

Our study assessed a number of immune cells and molecules beyond TILs, simultaneously in the primary breast tumor and involved and uninvolved axillary nodes and assessed the impact of these findings on patient clinical outcomes. It was of interest that we found consistent absence of GAL‐9+ tumor cells in the breast or involved nodes of relapsed patients, while GAL‐9+ tumor cells were seen in the controls. Collectively, published data suggest GAL‐9 to be an important molecule that should be further evaluated, taking into account the presence or absence of other checkpoint proteins in the tumor microenvironment.

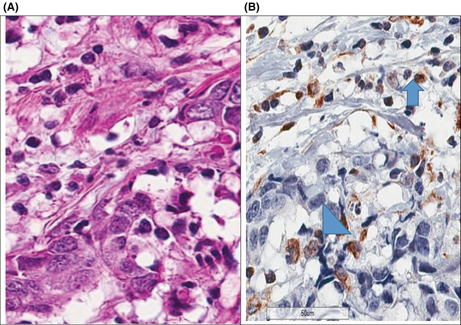

## 1 INTRODUCTION

Despite improved survival rates for early breast cancer (EBC) patients in the era of a multidisciplinary team approach, optimal locoregional and contemporary systemic treatments, patients with luminal B breast cancer (i.e., hormone receptor positive with high proliferation with or without HER2‐amplification) continue to have higher rates of relapse and poorer EBC‐specific survival when compared with luminal A disease.^1^, ^2^ Further, data from the Surveillance, Epidemiology, and End Results registry show that hormone receptor positive, HER2‐negative, or positive breast cancer accounts for 83% of those patients with known receptor status reported in 2010.^3^ Studies have consistently demonstrated a prognostic role of immune cells, most frequently in regard to tumor infiltrating lymphocytes (TILs) in triple negative and HER2‐positive breast cancer.^4^, ^5^ In contrast, the role of TILs and other immune cells in the tumor micro‐environment of patients with luminal B breast cancer is less certain. Given the relationship that exists between tumor cells, T cells, macrophages, and other immune cells, in the primary tumor and nodal region, a broader evaluation of the immune cells beyond TILs alone may be more informative.

The primary aim of the present study was to assess for the expression of immune biomarkers and their potential prognostic association with risk of recurrence in luminal B EBC patients. Specifically, we assessed for the presence of molecular markers of immune cells that function as suppressor or effector T cells and macrophages in the breast and axillary lymph nodes, in a population of luminal B EBC patients who had experienced an invasive breast cancer event, to assess whether the presence of one or more immune biomarker provided independent prognostic information.

## 2 MATERIALS AND METHODS

**FIGURE 1:**
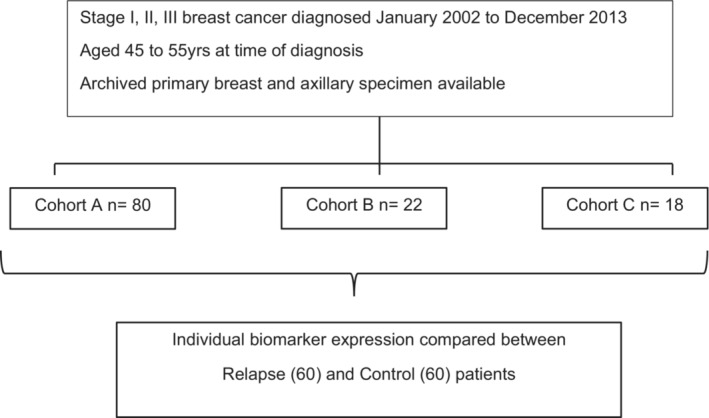
Study population.

A large clinical and pathological dataset which collected data prospectively since 1999 was interrogated to identify patients who fulfilled the inclusion criteria of luminal B disease (LB) and who had experienced an invasive breast cancer event (relapsed patient, R). As luminal B BC include patients with differing hormone receptor and/or HER2 receptor expression, we defined our study population as having LB disease in line with Cheang et al.^6^ Patients were categorized into three cohorts for purposes of illustrating the histopathological features in defining the study population (i) Cohort A—invasive ductal or mixed ductal‐lobular carcinoma which is grade 2 or 3 or Ki67 ≥ 14%, estrogen (ER^+^) and progesterone receptor positive (PR^+^) (defined as >1% nuclear staining) and HER2‐negative (HER2^−^); (ii) Cohort B—any grade tumor, ER^+^, PR^−^ and HER2^−^ and Ki67 ≥ 14%; and (iii) Cohort C—any grade tumor, ER^+^ or PR^+^ and HER2‐positive (HER2^+^). All patients in the entire study were then compared to age and stage‐matched patients treated in the same era who remain disease‐free (control patients, C; Figure 1). In line with our goal of assessing for immune markers which may impact on prognostic outcome, we did not analyze outcomes between R and C patients within the three cohorts separately, but as a collective group of patients with luminal B breast cancer. A breast cancer event was defined as a biopsy‐proven invasive breast cancer recurrence (locoregionally and/or metastatic site) or unequivocal metastatic disease on imaging. Key inclusion criteria included patients diagnosed with stage I, II, or III BC aged between 45 and 55 years at time of EBC diagnosis. Patients who were noncompliant with treatment recommendations, lost to follow‐up or who had co‐morbidities which impact on immune function were excluded.

Samples were obtained from archived formalin‐fixed paraffin embedded (FFPE) primary breast tumor with adjacent normal tissue and from involved and uninvolved ipsilateral axillary nodes from both groups of patients at the time of definitive surgery. Eight 4‐micron sections were cut from selected FFPE blocks. All slides were placed into a slide heater at 60° C for 20 mins. One slide each was then stained with hematoxylin and eosin (H&E), or with antibodies directed against CD68 (to detect macrophages), and the checkpoint molecules Cytotoxic T‐lymphocyte Antigen‐4 (CTLA‐4), Galectin‐9 (Gal‐9), Programmed death‐1 (PD‐1), and its ligand, PD‐L1.

All slides for immunohistochemistry (IHC) were stained on a Ventana Benchmark Ultra using Ventana Optiview as the chromagen and a light hematoxylin as the counterstain. After antigen retrieval, pretreatment sections were incubated with the primary antibodies (Table S1) for antigens, dilution factors, and incubation times. Staining was visualized using Ventana's OptiView 3,3‐diaminobenzidine (DAB) IHC Detection Kit, which is an indirect, biotin‐free system for detecting mouse IgG, mouse IgM and rabbit primary antibodies. After completion of staining, all immunohistochemistry slides were washed and dehydrated in 100% ethanol, cleared in xylene and mounted using DPX.

TILs were assessed on H&E‐stained tumor sections using a standardized evaluation approach recommended by an International TILs Working Group 2014.^7^ Briefly, one section (4–5 μm, magnification 40x) per patient was used. TILs in the stromal compartment were evaluated within the borders of the invasive tumor, with TILs outside of the tumor border excluded. Polymorphonuclear leukocytes were also excluded. Tumor hotspots were not targeted, but were included in the overall count, as long as they were contiguous with the field counted. TILs were reported as a percentage of stromal cells. Lymphocytes within a total of 500 cells were counted by three pathologists blinded to clinical data, and the results averaged.

For PD‐1, the number of PD‐1 positive lymphocytes in the tumor stroma, and in lymph nodes, was counted by the same three pathologists using magnification 200–400 times. Individual positive cells were counted over at least a 500 cell count, to ensure uniform calculations.

Similarly, PDL‐1, GAL‐9, CD68, and CTLA‐4 positive lymphocytes in tumor stroma and lymph nodes were counted using magnification 200–400 times providing a percentage of lymphocytes in stroma. PDL‐1, GAL‐9, CD68, and CTLA‐4 positive tumor cells were also calculated out of at least 500 tumor cells, providing a percentage of positive cells per tumor cells. The immune biomarkers were examined in two ways. We assessed the relationship between biomarker‐positive lymphocytes and adjacent stromal cells, with the data shown as the percentage of biomarker‐positive lymphocytes of the total cells in the selected region (A). We also examined the proportion of tumor cells that were positive for any of the biomarkers as a percentage of all tumor cells seen (B).

The study was approved by the Bellberry Limited and Curtin University Human Ethics and Research Committees (approval numbers 2015–03‐151 and HR 107/2015) and conducted in line with the National Statement on Ethical Conduct in Human Research 2007 and in accordance with the 2008 Declaration of Helsinki. Patients included in the study provided written consent for the use of their de‐identified medical information, as well as to the pathological examination of archived breast cancer and axillary nodal tissues.

### 2.1 Statistical analyses

Demographic, histopathological features and treatment details were described in mean and standard deviation (*SD*), or median and interquartile range (if variables were continuous and skewed), or frequency and percentage (if variable was categorical), by relapse status. Differences were assessed by independent samples t‐tests (or Mann–Whitney *U*‐tests when *t*‐test assumptions were violated) or chi‐squared tests (or Fisher's exact tests when chi‐squared test assumptions were violated). Demographic, histopathological features and adjuvant treatment administered to these three cohorts were reported and compared using either one‐way ANOVA (or Kruskal–Wallis test when ANOVA assumptions were violated) or chi‐squared tests (or Fisher's exact tests). Association between the biomarkers and breast cancer status (relapse/control) was assessed using logistic regression models adjusted for age, cohort, tumor status, lymph node status, progesterone status, and HER2 receptor status. All analyses were performed using StataIC/14.2 (StataCorp, Texas).

## 3 RESULTS

One hundred and twenty LB patients (R 60, C 60) were seen over the study period of January 2000 to June 2013. The mean age of the total population was 49.8 years with a median time from EBC diagnosis to death or last review of 83.3 months (95% CI 75.1, 94,4, Table 1).

**TABLE 1:** Patient and tumor characteristics

	Total (*n* = 120)	Relapse (*n* = 60)	Control (*n* = 60)	*p*‐values^a^
Age in years (mea*n* ± *SD*)	49.8 ± 3.4	50.6 ± 3.8	49.1 ± 2.9	**0.016**
EBC disease‐free (years)		‐	7.1 ± 2.1	
Relapse time to event (years)		5.0 ± 2.7	‐	
Disease presentation	*n* (% within group)			0.999
A: Grade 2 or 3, ER and PR positive, HER2 negative	80 (67)	40 (67)	40 (67)	
B: Any grade, ER positive and PR negative and HER2 negative	22 (18)	11 (18)	11 (18)	
C: Any grade, ER or PR positive and HER2 positive	18 (15)	9 (15)	9 (15)	
Deceased status				<0.001
Alive	79 (66)	19 (32)	60 (100)	
Deceased	41 (34)	41 (68)	0	
Primary EBC lymph node status				0.699
Negative	40 (33)	19 (32)	21 (35)	
Positive	80 (67)	41 (68)	39 (65)	
Site of first breast cancer event				‐
Metastatic	44 (73.3)	44 (73.3)	n/a	
Local recurrence	11 (18.3)	11 (18.3)	n/a	
Contralateral breast cancer	5 (8.3)	5 (8.3)	n/a	
Estrogen receptor (ER) status				0.999
Negative	2 (2)	1 (2)	1 (2)	
Positive	118 (98)	59 (98)	59 (98)	
Progesterone receptor (PR) status				0.648
Negative	24 (20)	13 (22)	11 (18)	
Positive	96 (80)	47 (78)	49 (82)	
HER2 receptor status				0.999
Negative	102 (85)	51 (85)	51 (85)	
Positive	18 (15)	9 (15)	9 (15)	
Tumor status				0.177^b^
T1	47 (39)	22 (37)	25 (42)	
T2	55 (46)	25 (42)	30 (50)	
T3	17 (14)	12 (20)	5 (8)	
T4	1 (1)	1 (1)	0	
Lymph node status				0.968^b^
N0	40 (33)	19 (32)	21 (35)	
N1	61 (51)	31 (52)	30 (50)	
N2	18 (15)	9 (15)	9 (15)	
N3	1 (1)	1 (1)	0	

*Note*: Bold indicates statistical significant value (*p* < 0.05).

^a^ Comparisons between relapse and control were performed using independent samples t‐test or chi‐squared test unless specified.

b Fisher's exact test was used.

Key characteristics were compared between R and C patients. Despite restricting patients for inclusion to those aged between 45 and 55 years at the time of breast cancer diagnosis, there remained a significant age difference between R and C, with the former being slightly older (*p* = 0.016, Table 1). The patients were otherwise well‐matched with respect to tumor size, nodal involvement, and receptor status for estrogen, progesterone, and HER2, and receipt of adjuvant chemotherapy and/or endocrine treatment. (Table 2).

**TABLE 2:** Patient, treatment, and disease outcome characteristics by cohort

			Total (*n* = 120)	Cohort A (*n* = 80)	Cohort B (*n* = 22)	Cohort C (*n* = 18)		*p*‐values^a^
Age in years (mean ± *SD*)			49.8 ± 3.4	49.8 ± 3.3	50.1 ± 3.4	49.6 ± 4.1		0.909
EBC disease‐free (years)			7.1 ± 2.1	7.3 ± 2.3	6.2 ± 1.2	7.5 ± 2.2		0.273
Relapse time to event (years)			5.0 ± 2.7	5.3 ± 2.6	4.0 ± 3.0	5.0 ± 2.6		0.364
Deceased status, *n* (%)								0.732
Alive			79 (66)	51 (64)	16 (73)	12 (67)		
Deceased			41 (34)	29 (36)	6 (27)	6 (33)		
Primary EBC lymph node status, *n* (%)								0.185
Negative			40 (33)	28 (35)	4 (18)	8 (44)		
Positive			80 (67)	52 (65)	18 (82)	10 (56)		
Site of first EBC event, *n* (%)								0.078^b^
Metastatic			44 (73.3)	31 (77.5)	5 (46)	8 (89)		
Local recurrence			11 (18.3)	7 (17.5)	4 (36)	0		
Contralateral breast cancer			5 (8.3)	2 (5)	2 (18)	1 (11)		
Estrogen receptor (ER) status, *n* (%)								0.311
Negative			2 (2)	1 (1)	0	1 (6)		
Positive			118 (98)	79 (99)	22 (100)	17 (94)		
Progesterone receptor (PR) status, *n* (%)								<0.001
Negative			24 (20)	0	19 (86)	5 (28)		
Positive			96 (80)	80 (100)	3 (14)	13 (72)		
Tumor status, *n* (%)								0.287^b^
T1			47 (39)	30 (38)	10 (45)	7 (39)		
T2			55 (46)	37 (46)	7 (32)	11 (61)		
T3			17 (14)	12 (15)	5 (23)	0		
T4			1 (1)	1 (1)	0	0		
Lymph node status, *n* (%)								0.013^b^
N0			40 (33)	28 (35)	4 (18)	8 (44)		
N1			61 (51)	45 (56)	10 (45)	6 (33)		
N2			18 (15)	6 (8)	8 (36)	4 (22)		
N3			1 (1)	1 (1)	0	0		
Adjuvant systemic treatment^c^, *n* (%)								

	Total (*n* = 120)		Cohort A (*n* = 80)		Cohort B (*n* = 22)		Cohort C (*n* = 18)	
	C^c^	R^c^	C^c^	R^c^	C^c^	R^c^	C^c^	R^c^
Chemotherapy								
No	7 (12)	4 (7)	7 (18)	4 (10)	0	0	0	0
Yes	53 (88)	56 (93)	33 (83)	36 (90)	11 (100)	11 (100)	9 (100)	9 (100)
	*p* = 0.343		*p* = 0.330		N/A		N/A	
Endocrine therapy								
No	1 (2)	5 (8)	0	4 (10)	0	1 (9)	1 (11)	0
Yes	59 (98)	55 (92)	40 (100)	36 (90)	11 (100)	10 (91)	8 (89)	9 (100)
	*p* = 0.207^b^		*p* = 0.116^b^		*p* = 0.999^b^		*p* = 0.999^b^	

*Note*: Cohort A: Grade 2 or 3, ER^+^PR^+^HER2^−^; Cohort B: Any grade, ER^+^PR^−^HER2^−^; Cohort C: Any grade, ER^+^ or PR^+^ and HER2^+^.

^a^ Comparisons across groups were performed using one‐way ANOVA or chi‐squared tests unless specified.

^b^ Fisher's exact test was used.

^c^ Chemotherapy included Anthracycline ± Taxane; Taxane only; Endocrine therapy included Tamoxifen or aromatase inhibitor or OA; Tamoxifen + aromatase inhibitor; C: control; R: relapse.

### 3.1 Clinical features

Key clinical characteristics of the three cohorts, that is, A (ER^+^PR^+^HER‐2^−^), B (ER^+^PR^−/+^HER2^−^) and C (ER^+^/PR^+/−^HER‐2^+^) are shown in Table 2. Cohort B patients experienced a shorter time to disease relapse albeit not of statistical significance. While a lower proportion of cohort B patients were deceased (27%) in the study period relative to cohorts A (36%) and C (33%), no statistical significance was observed. There was a trend for Cohort B patients having higher frequency of locoregional recurrence as their first event (*p* = 0.078). Tumor characteristics that were significantly associated with relapse included PR^+^, HER‐2^+,^ and lymph node involvement status (Table 2).

Overall, adjuvant chemotherapy and endocrine therapy were administered to 91% and 95% of the total population, respectively. Chemotherapy was not given to a greater proportion of cohort A C as compared to R (18% vs. 10%); while all patients in cohort B and C received adjuvant chemotherapy. Adjuvant endocrine treatment was given to 100%, 100%, and 89% of C across cohorts A, B, and C, respectively. This compared to 90%, 91%, and 100% of R across the three cohorts, respectively. At the time of data analysis, 41 (68%) patients who had experienced a breast cancer relapse had died.

### 3.2 Immune environment

We examined the immune environment of the tumor samples using immunohistochemistry for TILs and key checkpoint molecules expressed by T cells, that is, CTLA‐4A and PD‐1, as well as CD68^+^ macrophages and checkpoint molecules that can be expressed by macrophages, other innate immune cells, tissue and tumor cells, that is, PDL‐1 and GAL‐9A (Table 3). The analysis demonstrated that the immune profile in LB tumors varied slightly between individuals. For example, proportion of TILs ranged from 12.2 (interquartile range ± 13.1) and 17.7 (interquartile range ± 19.9), respectively, for R and C patients, and CD68^+^ macrophage ranged from 15.6 (interquartile range ± 11.2) and 17.9 (interquartile range ± 14.1), respectively, for R and C patients. A similar variation was seen for the checkpoint molecule positive lymphocytes and tumor cells. The large intra‐patient variability was also seen for involved and uninvolved lymph nodes with no statistically significant differences seen between the percentage of cells positive for each molecule between R compared to C (Table 3). There was an upward trend of greater numbers of TILs seen in primary tumors of C (*p* = 0.076).

**TABLE 3:** Descriptive statistics of biomarkers among all participants by relapse status

	mean ± SD *or* median (interquartile range)			
	Total	Relapse	Control	*p*‐value^a^
Ki67	35.0 ± 18.2 [*n* = 120]	36.3 ± 20.2 [*n* = 54]	33.7 ± 16.0 [*n* = 55]	0.454
Biomarkers in breast				
TILs	14.9 ± 17.0 [*n* = 120]	12.2 ± 13.1 [*n* = 60]	17.7 ± 19.9 [*n* = 60] Upward trend	0.076
PD1	3.0 ± 4.7 [*n* = 120]	2.8 ± 3.0 [*n* = 60]	3.3 ± 5.4 [*n* = 60]	0.566
PDL‐1A	0 (20) [*n* = 120]	0 (11.5) [*n* = 60]	0 (7.5) [*n* = 60]	0.676
PDL‐1B	0 (7.5) [*n* = 120]	0 (5) [*n* = 60]	0 (1) [*n* = 60]	0.878
GAL‐9A	5.8 ± 9.0 [*n* = 120]	6.8 ± 10.8 [*n* = 60] upward trend	4.8 ± 6.8 [*n* = 60]	0.221
GAL‐9B	1.8 ± 2.7 [*n* = 120]	0 (5.5) [*n* = 60]	1.9 ± 2.5 [*n* = 60]	0.518
CD68	16.7 ± 12.8 [*n* = 119]	15.6 ± 11.2 [*n* = 60]	17.9 ± 14.1 [*n* = 59]	0.316
CTLA‐4A	0 (12.5) [*n* = 120]	0 (5) [*n* = 60]	0 (10) [*n* = 60]	0.718
CTLA‐4B	0 (21.5) [*n* = 120]	0 (10) [*n* = 60]	0 (20) [*n* = 60]	0.672
Biomarkers in involved lymph node				
PD1	3.3 ± 3.2 [*n* = 59]	3.7 ± 3.6 [*n* = 34] upward trend	2.8 ± 2.6 [*n* = 25]	0.272
PDL‐1A	1.8 ± 2.4 [*n* = 59]	1.8 ± 2.5 [*n* = 34]	1.8 ± 2.4 [*n* = 25]	0.944
PDL‐1B	0.5 ± 1.1 [*n* = 59]	0.5 ± 1.3 [*n* = 34]	0.5 ± 0.9 [*n* = 25]	0.947
GAL‐9A	2.9 ± 3.8 [*n* = 59]	2.9 ± 3.9 [*n* = 34]	2.9 ± 3.7 [*n* = 25]	0.983
GAL‐9B	0 (4) [*n* = 59]	0 (4) [*n* = 34]	1.2 ± 1.4 [*n* = 25]	0.650
CD68	16.8 ± 13.5 [*n* = 59]	17.2 ± 12.4 [*n* = 34]	16.2 ± 15.1 [*n* = 25]	0.786
CTLA‐4A	0 (4) [*n* = 59]	0 (1) [*n* = 34]	0.4 ± 1.3 [*n* = 25]	0.659
CTLA‐4B	0 (13) [*n* = 59]	0 (6) [*n* = 34]	0 (2) [*n* = 25]	0.470
Biomarkers in uninvolved lymph node				
PD1	4.2 ± 4.4 [*n* = 73]	3.5 ± 3.1 [*n* = 35]	4.7 ± 5.4 [*n* = 38]	0.254
PDL‐1A	2.8 ± 4.0 [*n* = 73]	2.7 ± 3.2 [*n* = 35]	2.8 ± 4.7 [*n* = 38]	0.890
GAl‐9A	3.3 ± 5.2 [*n* = 72]	0 (10) [*n* = 35]	3.1 ± 4.7 [*n* = 37]	0.386
CD68	19.9 ± 12.0 [*n* = 73]	19.8 ± 10.3 [*n* = 35]	20.0 ± 13.6 [*n* = 38]	0.956
CTL4‐A	0 (4) [*n* = 73]	0 (2) [*n* = 35]	0 (1) [*n* = 38]	0.886

a Comparisons between relapse and controls were performed using independent samples *t*‐test or Mann–Whitney *U*‐test.

Overall, expression of PD1 was similar between R and C, with low levels seen in breast and nodal specimens. There was uniform absence of CTLA4 expression in both lymphocytes and tumor cells of R and C, with a mean of <1% staining seen in involved nodes of C.

**FIGURE 2:**
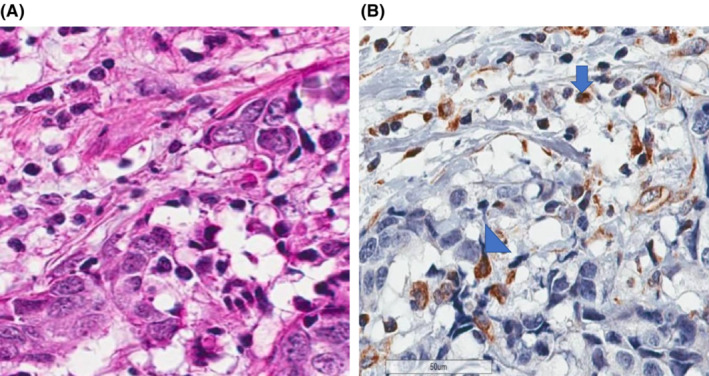
(A) H&E of primary breast cancer specimen of a control patient demonstrating positivity on breast cancer cells and stromal lymphocytes (x40, respectively). (B) GAL9 immunohistochemistry stain in primary breast cancer specimen of a control patient demonstrating positivity on breast cancer cells (GAL‐9A) (arrowhead) and (stromal lymphocytes) GAL9‐B (arrow) (x40, respectively; scale bar, 50 μm).

The evaluation of GAL‐9 (GAL‐9A) in the primary tumor did not demonstrate statistically significant differences between R and C patients, but numerical differences were seen. It was more frequently expressed on tumor cells and stromal lymphocytes in C. In R, GAL‐9 was not expressed by tumor cells but was seen in stromal lymphocytes (GAL‐9B) (Figure 2). There were no other notable differences in the immune profile of R and C (Table 3).

**FIGURE 3:**
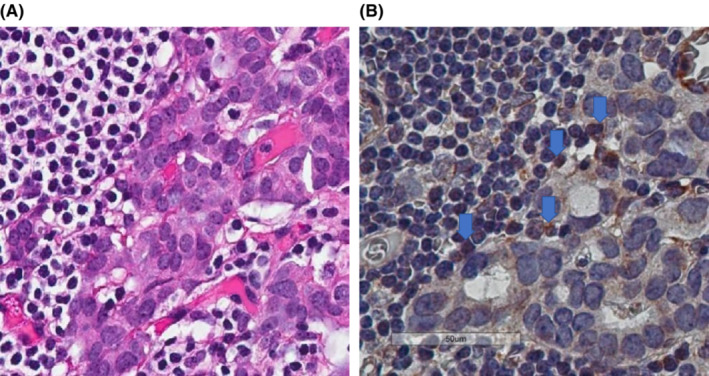
(A) H&E stain of involved lymph node from a relapsed patient where breast cancer cells (GAL‐9A) was negative and (stromal lymphocytes) GAL‐9B was positive (x40, respectively). (B) GAL9 immunohistochemistry stain of involved lymph node from a relapsed patient where breast cancer cells (GAL‐9A) was negative and (stromal lymphocytes) GAL‐9B was positive (blue arrows) (x40, respectively; scale bar, 50 μm).

An examination of involved lymph nodes from R and C did not reveal any statistically significant differences, although a few trends were noted. Involved lymph nodes in R did not contain GAL‐9^+^ tumor cells (i.e., GAL‐9B), although GAL‐9^+^ lymphocytes (i.e., GAL‐9A) were seen (Table 3 and Figure 3). However, the proportion of GAL‐9^+^ lymphocytes in involved lymph nodes were similar in those patients who relapsed or remained disease‐free. There was an upward trend of CD68^+^ macrophage and PD‐1^+^ and CTLA‐4^+^ tumor cells in the involved lymph nodes of R compared to C.

Similar to involved lymph nodes, a comparison of immune cells in uninvolved lymph nodes from R versus C did not reveal any statistically significant differences. Of interest, evaluation of GAL‐9 in uninvolved lymph nodes demonstrated no expression of GAL‐9 on lymphocytes in R, while GAL‐9^+^ lymphocytes were seen in C (Table 3).

Although differences did not reach statistical significance, there did appear to be biomarker expression differences in the breast and involved lymph node in individual patients from a given cohort. T‐cell checkpoint molecules (PD‐1 and CTLA‐4) were generally not expressed in cohort A which was the patient population with the longest interval to breast cancer relapse (Table 4). Cohort B patients had the shortest disease‐free interval, and all biomarkers appeared to be present in breast tumor cells, involved and uninvolved lymph nodes (Table 4). As Ki67 greater than 14% was an inclusion criterion, we found no association between the magnitude of Ki67 with the risk of relapse.

**TABLE 4:** Descriptive of biomarkers among patients by disease cohort

	mean ± *SD or* median (interquartile range)				
	Total	Cohort A	Cohort B	Cohort C	*p*‐value^a^
Ki67	35 ± 18.2 [*n* = 120]	33.8 ± 16.6 [*n* = 80]	36.0 ± 23.1 [*n* = 22]	44.9 ± 17.7 [*n* = 7]	0.296
Biomarkers in breast					
TILs	14.9 ± 17.0 [*n* = 120]	14.4 ± 15.7 [*n* = 80]	14.8 ± 18.7 [*n* = 22]	17.3 ± 21.3 [*n* = 18]	0.813
PD1	3.0 ± 4.7 [*n* = 120]	3.3 ± 5.2 [*n* = 80]	3.2 ± 2.7 [*n* = 22]	0 (2.5) [*n* = 18]^b^	0.020
PDL‐1A	0 (20) [*n* = 120]	0 (14) [*n* = 80]^b^	1.8 ± 3.2 [*n* = 22]	0 (2.5) [*n* = 18]^b^	0.654
PDL‐1B	0 (7.5) [*n* = 120]	0 (5) [*n* = 80]^b^	0.8 ± 1.9 [*n* = 22]	0.1 ± 0.2 [*n* = 18]	0.594
GAL‐9A	5.8 ± 9.0 [*n* = 120]	0 (27.5) [*n* = 80]^b^	5.2 ± 6.4 [*n* = 22]	7.2 ± 9.5 [*n* = 18]	0.549
GAl‐9B	1.8 ± 2.7 [*n* = 120]	1.9 ± 2.7 [*n* = 80]	1.7 ± 2.8 [*n* = 22]	1.6 ± 2.6 [*n* = 18]	0.930
CD68	16.7 ± 12.8 [*n* = 119]	16.1 ± 11.5 [*n* = 79]	20.0 ± 14.9 [*n* = 22]	15.4 ± 15.3 [*n* = 18]	0.415
CTLA‐4A	0 (12.5) [*n* = 120]	0 (10) [*n* = 80]^b^	0.6 ± 1.3 [*n* = 22]	0 (0.5) [*n* = 18]^b^	0.847
CTLA‐4B	0 (21.5) [*n* = 120]	0 (20) [*n* = 80]^b^	0 (1) [*n* = 22]^b^	0 (2.5) [*n* = 18]^b^	0.281
Biomarkers in involved lymph node					
PD1	3.3 ± 3.2 [*n* = 59]	3.3 ± 3.7 [*n* = 38]	3.4 ± 2.6 [*n* = 14]	3.1 ± 1.8 [*n* = 7]	0.982
PDL‐1A	1.8 ± 2.4 [*n* = 59]	1.4 ± 2.3 [*n* = 38]	2.5 ± 2.9 [*n* = 14]	2.4 ± 2.4 [*n* = 7]	0.302
PDL‐1B	0.5 ± 1.1 [*n* = 59]	0 (1) [*n* = 38]^b^	0.6 ± 0.9 [*n* = 14]	0.7 ± 1.1 [*n* = 7]	0.412
GAL‐9A	2.9 ± 3.8 [*n* = 59]	2.8 ± 3.9 [*n* = 38]	2.3 ± 2.6 [*n* = 14]	5.1 ± 4.7 [*n* = 7]	0.243
GAl‐9B	0 (4) [*n* = 59]	0 (3) [*n* = 38]^b^	1.2 ± 1.3 [*n* = 14]	1.6 ± 1.7 [*n* = 7]	0.301
CD68	16.8 ± 13.5 [*n* = 59]	16.8 ± 12.8 [*n* = 38]	14.2 ± 11.3 [*n* = 13]	21.4 ± 20.3 [*n* = 7]	0.523
CTLA‐4A	0 (4) [*n* = 59]	0 (1) [*n* = 38]^b^	0.8 ± 1.8 [*n* = 14]	0.9 ± 1.9 [*n* = 7]	0.826
CTLA‐4B	0 (13) [*n* = 59]	0 (3) [*n* = 38]^b^	1.9 ± 3.8 [*n* = 14]	3.1 ± 7.4 [*n* = 7]	0.923
Biomarkers in uninvolved lymph node					
PD1	4.2 ± 4.4 [*n* = 73]	4.3 ± 5.1 [*n* = 47]	4.3 ± 3.5 [*n* = 17]	3.4 ± 2.4 [*n* = 9]	0.687
PDL‐1A	2.8 ± 4.0 [*n* = 73]	2.6 ± 4.3 [*n* = 47]	3.4 ± 3.8 [*n* = 17] Upward trend	2.3 ± 2.3 [*n* = 9]	0.761
GAL‐9A	3.3 ± 5.2 [*n* = 72]	3.7 ± 5.5 [*n* = 47]	1.9 ± 2.5 [*n* = 16] Downward trend	3.6 ± 7.3 [*n* = 9]	0.489
CD68	19.9 ± 12.0 [*n* = 73]	19.5 ± 11.2 [*n* = 47]	23.5 ± 14.9 [*n* = 17]	15.6 ± 9.3 [*n* = 9]	0.259
CTLA‐4A	0 (4) [*n* = 73]	0 (2.5) [*n* = 47]^b^	0.4 ± 1.0 [*n* = 17]	2.2 ± 5.8 [*n* = 9]	0.856

a Comparisons across groups were performed using one‐way ANOVA or Krukal–Wallis test.

^b^ Mostly not expressed.

## 4 DISCUSSION

It has been shown that the presence of TILs in the stromal environment of primary breast tumors may be a key factor contributing to poorer outcomes in patients with triple negative and HER2‐positive breast cancer.^4^, ^5^, ^8^, ^9^ In contrast, the reports of the importance of TILs as a prognostic factor in estrogen receptor positive breast cancer are conflicted. Some studies demonstrate no impact on disease‐free or overall survival,^4^ while others have shown that the presence of high levels of TILs is a negative prognostic factor,^10^ subgroup analysis in a review of neoadjuvant trials suggests low TILs to be associated with better survival^11^ and still others report longer survival in patients with tumors shown to have high immune scores.^12^ Our results, though not significant, showed a trend in favor of higher TILs in the primary tumor of patients who remain disease‐free.

A possible explanation for these discordant results are the choice of immune cells that are studied, as well as the setting in which the patient population is being evaluated. The meta‐analysis by Mao et al included 25 studies in which intra‐tumoral and/or stromal TILs were evaluated. Only three studies in the meta‐analysis were prospective in nature, with the study period for patients studied ranging from 1997 to 2010. The marker used to identify TILs included CD8, CD4, CD20, FOXP3, B7‐H3, and PD‐1. Though the authors found CD8+ lymphocytes being associated with significantly improved and FOXP3+ lymphocytes associated with significantly reduced disease‐free survival for the total number of studies included, the hazard ratio for both breast cancer outcome measures was not significant in estrogen receptor positive patients.^4^ We defined TILs using CD8 positivity only and found slightly higher levels in the HER2‐positive cohort C patients, but essentially low to intermediate numbers of TILs in the study population overall (mean 14.9%) The review by Pellegrino et al suggests that identifying TILs by FOXP3 positivity and CTLA‐4 expression in both tumor cells and TILs was associated with worse disease‐free and overall survival. In a large cohort of patients treated in six neoadjuvant clinical trials undertaken by the German Breast Group, a retrospective analysis for TILs was conducted as part of the planned translational research program. The authors defined stromal TILs similarly to our study, in accordance with the International TILs Working Group, as a continuous measurement with low TILs (0%–10%), intermediate (11%–59%), and high (60%–100%). The luminal HER2‐negative subtype accounted for 37% of the collective total population, with proportion of low, intermediate, and high TILs seen in 56%, 32%, and 13%, respectively. Following a median follow‐up of 63 months, there was no significant association with TIL concentration and disease‐free survival in patients with luminal B, HER2‐negative disease. Of interest, there was a significantly longer survival in this group of patients in favor of a low TILs environment, even when adjusted for all baseline characteristics and achievement of a complete pathological response.^10^ It is noteworthy that in all six of these randomized clinical trial, patients were allocated to receive anthracycline chemotherapy in both the control and investigational arm. It has been suggested that the efficacy of anthracycline‐based adjuvant chemotherapy is as a result of enhanced immunogenic tumor cell death leading to up‐regulated tumor antigen cross‐presentation by dendritic cells (DCs). The latter can then lead to induction of tumor‐specific CD8^+^ cytotoxic T lymphocytes (CTL) in lymph nodes.^13^ This may be a possible mechanism of action that may be effective in LB breast cancer patients treated with adjuvant anthracycline chemotherapy and may be a factor influencing the interpretation of the baseline numbers of T‐lymphocytes and outcome.

A study of 566 LB tumor samples from The Cancer Genome Atlas (TCGA) and using the ESTIMATE (Estimation of Stromal and Immune cells in Malignant Tumour tissues Expression data) algorithm was undertaken to evaluate the tumor microenvironment in LB cancers to identity potential biomarkers to predict survival. By RNA sequencing, 175 patients were LB. The authors demonstrated a significant overall survival benefit (*p* = 0.015) in patients whose tumors expressed a high versus low expression ImmuneScore. No correlation with the StromalScore was seen in this patient population. The authors did not provide a separate analysis of the ImmuneScore for luminal A as compared to luminal B patients.^11^

In our study, we assessed for the role of macrophages as an immune predictor of outcome between relapsed and control patients, as macrophages may be the dominant immune cell in some tumor microenvironments. Their numerical dominance alongside their ability to adopt functional phenotypes ranging from potent suppressors (the most suppressive termed alternatively‐activated or M2 macrophages) to powerful pro‐inflammatory anti‐tumorigenic cells (the most potent termed M1 macrophages) could enable macrophages to play a greater role in breast cancer outcomes. Although we could identify similar numbers of macrophages in all three compartments of tissue that we examined, there were no significant differences between the breast, involved and uninvolved nodes of either relapsed or control patients. As macrophages can regulate T‐cell function via a number of mechanisms involving PD‐1 upregulation, further attention to evaluating this immune cell type as a prognostic variable in future research is important.

GAL‐9 is an immune checkpoint protein that can reduce pro‐inflammatory cells and increase anti‐inflammatory regulatory T cells.^14^ GAL‐9 ligates with T‐cell immunoglobulin and mucin protein‐3 (TIM‐3), a cell surface inhibitory receptor found mainly on activated CTLs and CD4^+^ T cells, as well as PD‐1 leading to T‐cell apoptosis thereby preventing T cell‐mediated tumor cell elimination.^15^, ^16^, ^17^ Preclinical data in MCF‐7 cells with high and low levels of GAL‐9 were evaluated with the demonstration of lower metastasizing potential in the former.^18^ In a study of 109 triple negative breast cancer patients whose primary breast tumor was evaluated for GAL‐9^+^ immune cells, the authors found high GAL‐9 was significantly associated with higher levels of stromal TILs, PD‐L1 positive tumor cells and negative PD‐L1 immune cells. In a subgroup of patients with PD‐L1 negative tumor cells, low GAL‐9 expression was associated with a significantly shorter overall survival.^19^ Much of the published data on GAL‐9 has focused on its role when expressed on tumor cells. We evaluated GAL‐9 expression both on tumor cells and lymphocytes in an attempt to identify whether the cell on which this check point protein was expressed differed. We acknowledge in this small study that there were no statistically significant differences in GAL‐9^+^ between patients who relapsed or remained disease‐free; though it was of interest that we found consistent absence of GAL‐9^+^ (GAL‐9B) tumor cells in the breast or involved nodes of relapsed patients, while GAL‐9^+^ tumor cells were seen in the controls. Collectively, published data suggest GAL‐9 to be an important molecule that should be further evaluated, taking into account the presence or absence of other checkpoint proteins in the tumor microenvironment.

In the current study, we were unable to demonstrate a significant immune biomarker which was significantly associated with a higher likelihood of an invasive breast cancer event. We recognize limitations in our research which included the inherent problems of the retrospective nature of the study. Although we restricted patient inclusion to those within the 45–55 years of age at time of diagnosis to avoid imbalance in age‐related decline in immune function,^20^ patients who experienced a relapse were significantly older than their control counterpart. The sample size calculation for our study was based on an anticipated 5% difference in one or more of the immune cell biomarkers between relapsed and control patients, and this difference was only just reached in the evaluation of TILs (difference of 5.5%), with all other biomarkers having ≤2% difference between the two patient groups. Thus, it is possible that a larger study may have provided more informative results. It is worthwhile to articulate some strengths of the current study which included prospectively collected data which confirms accuracy of the treatment details, and importantly outcome data of confirmed invasive breast cancer recurrence and known date of death or last follow‐up. In addition, we assessed a number of immune cells and molecules beyond just TILs, simultaneously in the primary breast tumor and involved and uninvolved axillary nodes—this approach being unique in our study design.

In conclusion, we believe that further research into the role of the immune status of tumor cells and its microenvironment is needed in this most common subtype of breast cancer. It is likely that within the heterogenous group of breast cancers termed luminal B, it will be possible to define those tumors where immune‐driven mechanisms play an important role in prognosis and possibly be a target for therapeutic intervention.

## AUTHOR CONTRIBUTIONS

**Arlene Chan:** Conceptualization (equal); data curation (equal); funding acquisition (equal); methodology (equal); project administration (equal); resources (equal); software (equal); supervision (equal); visualization (equal); writing – original draft (equal); writing – review and editing (equal). **Jespal Gill:** Conceptualization (equal); investigation (equal); methodology (equal). **HuiJun Chih:** Formal analysis (equal); validation (equal). **Delia Nelson:** Conceptualization (equal); investigation (equal); methodology (equal); writing – original draft (equal).

## Supporting information


Table S1.

## Data Availability

Data Availability StatementThe data that support the findings of this study are available on request from the corresponding author. The data are not publicly available due to privacy or ethical restrictions.
