# Supplementary material for: Prognostic role of immune environment in luminal B early breast cancer
Source: Cancer Med. 2023 Feb 7;12(7):8278–88. doi: 10.1002/cam4.5642 (PMC10134297; doi:10.1002/cam4.5642)
Supplement: Supplementary file 1 — Table S1. [file CAM4-12-8278-s001.docx]

**Supplementary Table 1: Antibodies used for Luminal B tumour sections**

| **Antigen targeted** | **Clone** | **Code #/Supplier** | **Antigen retrieval pre-treatment** | **Dilution** | **Incubation time** |
| --- | --- | --- | --- | --- | --- |
| CD68 | KPI mouse monoclonal | 168M-96  Cell Marque | HIER CCI 64 min | 1:1000 | 32 min |
| CTLA-4 (CD152) | Rabbit Polyclonal | PA5-26465  Thermofisher | HIER CCI 64 min | 1:200 | 64 min |
| Galectin-9 | Rabbit Polyclonal | PA5-32252  Thermofisher | HIER CCI 32 min | 1:500 | 32 min |
| PD-1 (CD279) | MRQ-22 mouse monoclonal | 315M-95 Cell Marque | HIER CCI 24 min | 1:500 | 20 min |
| PDL-1 | EIL3M(R) rabbit monoclonal | 13684 Cell Signaling Technology | HIER CCI 64 min | 1:200 | 64 min |

HIER = heat induced epitope retrieval
